# Vitamin D receptor isoform VDRA suppresses hepatocellular tumorigenesis by restricting YAP nuclear localization

**DOI:** 10.1038/s41419-026-08792-0

**Published:** 2026-04-29

**Authors:** Lizhi Li, Shiwei He, RuoYa Wang, Jianbin Wang, Ziyi Zheng, Zhe Wang, Bingyan Li, Weimin Ye, Ping Wang

**Affiliations:** 1 Department of Nutrition and Food Safety, School of Public Health, Fujian Medical University, Fuzhou, China; 2 Hepatobiliary and Pancreatic Center, Shengli Clinical Medical College of Fujian Medical University, Fuzhou, Fujian China; 3 Hepatobiliary and Pancreatic Center, Affiliated Provincial Hospital of Fuzhou University, Fuzhou, Fujian China; 4 NHC Key Laboratory of Etiological Epidemiology of Chronic Diseases with High Incidence in Fujian-Taiwan Area, Fujian Medical University, Fuzhou, China; 5 Institute of Population Medicine, School of Public Health, Fujian Medical University, Fuzhou, Fujian China; 6 Department of Nutrition and Food Hygiene, School of Public Health, Medical College of Soochow University, Suzhou, China

**Keywords:** Tumour-suppressor proteins, Target validation

## Abstract

Hepatocellular carcinoma (HCC) frequently arises from uncontrolled proliferation of hepatocyte epithelium, a process potently driven by the transcriptional regulator yes-associated protein (YAP). Although 1,25(OH)₂D₃, the active form of vitamin D, exerts established anti-proliferative effects through its receptor (VDR), the specific roles of its two principal isoforms, VDRA and VDRB1, have remained largely undefined. Clinical analysis of the TCGA-LIHC database identified high VDRB1 and low VDRA expression as robust independent risk factors for poor prognosis, suggesting their divergent impacts on HCC progression. Here, we demonstrated that 1,25(OH)₂D₃ induced coordinate expression of both VDR isoforms in HCC cells, activated divergent transcriptional programs, particularly concerning the regulation of YAP target genes. Crucially, functional overexpression assays revealed that VDRA, but not VDRB1, potently inhibited YAP activation, an effect synergistically augmented by combined treatment with 1,25(OH)₂D₃. This regulatory axis was further substantiated by endogenous VDR silencing, while the VDRA-mediated repression of YAP signaling was substantially abrogated by constitutively active YAP-5SA. Functionally, VDRA impaired the proliferation and colony-forming capacity of HCC cells—an inhibitory action significantly rescued by YAP-5SA overexpression—whereas VDR knockdown promoted cell growth. Mechanistically, VDRA efficiently translocated to the nucleus to restrict YAP nuclear localization, while 1,25(OH)₂D₃-treated VDRB1 formed perinuclear condensates that failed to enter the nucleus. To confirm these mechanisms, we employed patient-derived HCC organoids (validated by H&E, RNA-seq, and WES). Subcutaneous engraftment demonstrated that VDRA overexpression, but not VDRB1, profoundly suppressed tumor growth by attenuating YAP activity. Taken together, our findings establish the VDRA isoform as the primary mediator of 1,25(OH)₂D₃-induced tumor suppression in HCC. By demonstrating that VDRA curtails hepatocarcinogenesis specifically by restricting YAP nuclear localization, this study delineates a novel isoform-specific mechanism within the vitamin D signaling axis, nominating VDRA as a promising biomarker and therapeutic target for YAP-driven liver cancer.

## Introduction

Hepatocellular carcinoma (HCC) is ranked as the sixth most commonly diagnosed cancer and the fourth primary contributor to cancer-related mortality on a global scale [1, 2]. Despite the availability of diverse treatment options for HCC patients, the median survival rate continues to fall below two years, even when utilizing the most potent medical interventions [3–6]. Liver regeneration triggered by biological factors like fatty liver, alcohol consumption, viruses, and inflammation typically necessitates the proliferation of differentiated hepatocytes to offset cell loss [7]. Furthermore, the abnormal proliferation of hepatocytes is also a leading cause of HCC. The yes-associated protein (YAP) serves as a significant downstream effector of the Hippo pathway, which is known to exert crucial oncogenic roles in various types of human cancers, particularly in HCC [8, 9]. Overexpression of YAP facilitates the activation of TEAD-dependent transcription of the cell proliferation gene *CTGF*, thereby promoting tumor cell proliferation [10]. Thereinto, YAP is considered a potential therapeutic target implicated in the proliferation of HCC cells [11–13]. However, targeting transcriptional activator YAP directly poses technical challenges. Hence, it is crucial to identify additional strategies aimed at targeting YAP to improve the prognosis of patients with HCC.

The biologically activated form of vitamin D [1,25(OH)_2_D_3_] or its analogs bind to the vitamin D receptor (VDR) to transcriptionally regulate gene expression, thereby eliciting VDR’s physiological functions. Several studies have highlighted the potential therapeutic advantages of 1,25(OH)_2_D_3_ or its analogues in the treatment of breast cancer [14, 15], colon cancer [16–18], and prostate cancer [19, 20]. Moreover, recent research unveiled that vitamin D activity appears to potentiate immune responses to cancer [21]. The serum levels of 25-hydroxyvitamin D_3_ [25(OH)D_3_], which are synthesized by 25-hydroxylases (CYP2R1 and CYP27A1) in the liver, exhibited an inverse correlation with the susceptibility to HCC [22–24]. Additionally, the level of bioavailable 25(OH)D_3_ was found to be significantly correlated with improved survival in patients with HCC [25, 26]. Previous research has shown that 1,25(OH)_2_D_3_ notably augmented the sensitivity of HCC to chemotherapeutic agents, consequently curbing the proliferation of HCC [27–30]. Specifically, blocking the binding of 1,25(OH)_2_D_3_ to VDR exacerbates the advancement of HCC [31]. Nonetheless, other studies indicated that 25(OH)D_3_ may potentially elevate the risk of developing HCC, and 1,25(OH)_2_D_3_ does not restrain the proliferation of HCC [32, 33]. The underlying mechanism responsible for the conflicting results remains ambiguous. This leads to the postulation that 1,25(OH)_2_D_3_ possesses the capacity to affect HCC in various ways, particularly when considering the lower VDR expression in the surrounding hepatic parenchymal cells *(*https://www.proteinatlas.org*/ENSG00000111424-VDR/tissue+cell+type/live*r).

The VDR, functioning as both a transcription factor and a receptor, comprises eight transcripts. Among these, the most abundantly expressed transcripts are translated into VDRA and VDRB1 (https://www.ncbi.nlm.nih.gov/gene/7421). Notably, VDRB1 possesses a unique 50-amino-acid N-terminal extension compared to VDRA, yet its biological significance remains largely underexplored [34]. Emerging evidence suggests that VDRB1 exhibits distinct, organ-specific ligand sensitivity [34–36], and our recent work has demonstrated that these two isoforms exert divergent prognostic impacts on patients with renal cell carcinoma [37].

Despite established reports of VDR–YAP crosstalk in various cancers, the literature presents a functional paradox, with Vitamin D signaling showing both stimulatory and inhibitory effects on YAP activity. We hypothesized that this functional heterogeneity might be rooted in isoform-specific signaling cascades. In this study, we demonstrate that VDRA, but not VDRB1, significantly potentiates the anti-proliferative effects of 1,25(OH)_2_D_3_ by suppressing YAP activation in both HCC cell lines and patient-derived organoids. By identifying this “isoform switch,” our work provides a mechanistic resolution to the previously documented discrepancies in VDR–YAP interactions and highlights VDRA as a precise, actionable target for countering YAP-driven hepatocarcinogenesis.

## Results

### 1,25(OH)₂D₃ upregulates two major isoforms of VDRA and VDRB1 in HCC Cells

**Fig. 1:**
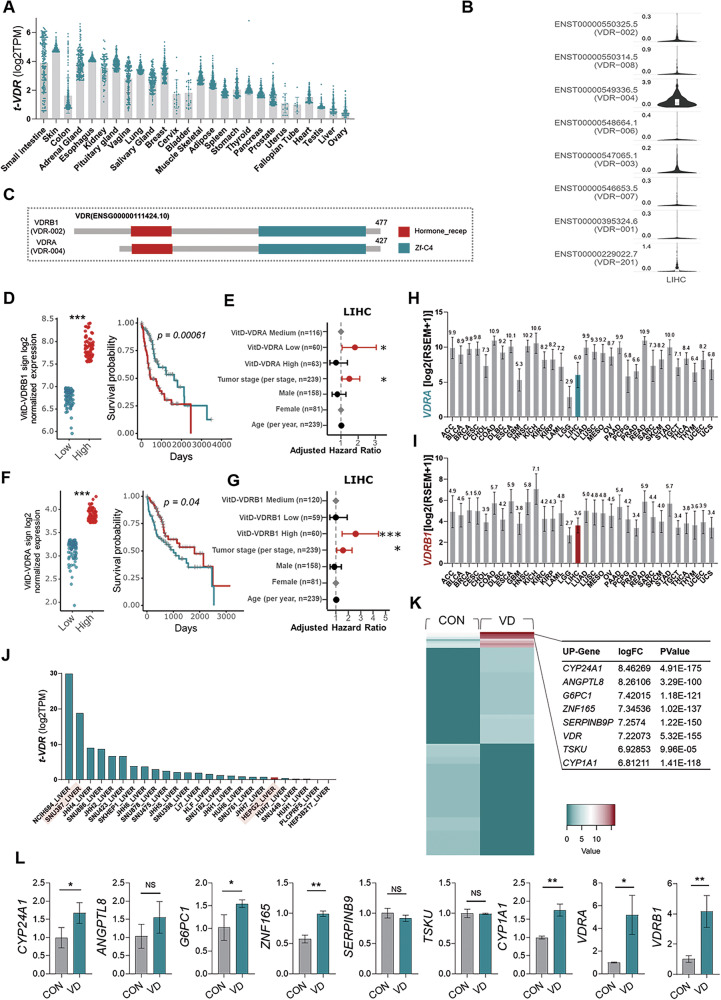
Both VDRA and VDRB1 were induced by 1,25(OH)_2_D_3_ in HepG2 cells. **A** Total expression of vitamin D Receptor (t-VDR) in diverse human tissues; **B** The levels of VDR transcripts in HCC; **C** The structure of VDR isoforms VDRA and VDRB1; **D** Patient stratification based on VDRB1 expression levels (left) and corresponding Kaplan-Meier survival analysis (right), showing that high VDRB1 expression correlates with significantly shorter overall survival; **E** Multivariate Cox regression analysis identifying high VDRB1 expression as a robust independent risk factor for mortality; **F** Patient stratification by VDRA expression (left) and survival analysis (right), revealing that low VDRA levels are associated with poor prognosis. **G** Multivariate Cox regression identifying low VDRA expression as an independent risk factor; **J** The expression of t-VDR in all cell lines of HCC; **H**, **I** *VDRA* or *VDRB1* expression in various types of tumors; **G** The expression of *t-VDR* in all cell lines of HCC; **K** Transcriptome sequencing results of HepG2 cells treated with or without 1,25(OH)_2_D_3_; **L** RT-qPCR was conducted to detect VDR target genes using three biological replicates; All mRNA expressions were normalized with *β-actin*; Subsequent statistical analysis was performed with unpaired two-sided student t-tests or one-way ANOVA with Tukey’s method for multiple comparisons. NS present not statistically significant; *p < 0.05, **p < 0.001, ***p < 0.0001. VD, 1,25(OH)_2_D_3_.

Although the total VDR expression was discernibly lower in the liver compared to other organs (Fig. 1A), eight VDR transcripts were still detected within HCC (Fig. 1B). Among these, *VDR-002* and *VDR-004* were the most abundant, corresponding to the translated forms VDRB1 and VDRA, respectively (Fig. 1C). Clinical analysis using the TCGA-LIHC database revealed that VDRB1 and VDRA exert opposing impacts on patient outcomes. Specifically, elevated VDRB1 expression correlated with significantly shorter overall survival (*p* = 0.00061; Fig. 1D), whereas reduced VDRA levels were associated with a poorer prognosis (*p* = 0.04; Fig. 1E). Multivariate Cox proportional hazards analysis, adjusted for age, sex, and tumor stage, further identified high VDRB1 and low VDRA expression as robust independent risk factors for mortality in HCC patients (aHR > 1, *p* < 0.05; Fig. 1F, G). These clinical findings suggest that the VDRA/VDRB1 balance is a critical determinant of HCC progression and patient survival.

Notably, although the expression of both VDRA and VDRB1 exhibited a marked decline in HCC compared to other tumor types, VDRA levels remained consistently higher than those of VDRB1 (Fig. 1H, I). The expression of total *VDR* was minimal in HCC cells, especially within HepG2 cells (Fig. 1J). Hence, we posed the question of whether HepG2 possesses the capacity to respond to 1,25(OH)_2_D_3_ administration. Transcriptome sequencing divulges that both *CYP24A1*, *NAGPL8, G6PC1, ZNF165, VDR, SERPINB9, TSKU* and *CYP1A1* were identified as top 8 up-regulated genes following treatment with 1,25(OH)_2_D_3_ (Fig. 1K). RT-qPCR was employed to validate the top 8 up-regulated genes resulting from treatment with 1,25(OH)_2_D_3_ as depicted in Fig. 1L. The data disclosed that HepG2 cells treated with 1,25(OH)_2_D_3_ showed an increase in the expression of *CYP24A1*, *G6PC1, ZNF165*, and *CYP1A1*. Moreover, either VDRA or VDRB1 in HepG2 cells was upregulated by 1,25(OH)_2_D_3_ (Fig. 1L). This observation leads to the postulation that HepG2 cells possess an intact vitamin D system and both VDRA and VDRB1 were induced by 1,25(OH)_2_D_3_ in HCC cells Table 1.

**Table 1:** Primer sequences of RT-PCR.

Gene	Forword (5‘-3‘)	Reverse (5‘-3‘)
*VDRA*	TGGAGACTTTGACCGGAACG	GGGCAGGTGAATAGTGCCTT
*VDRB1*	CGGCTGCGAGCATTAGAG	CTTCAGACCCAAAGGCTTCCT
*CYP1A1*	GCGCTATGACCACAACCACC	AAACCCAGCTCCAAAGAGGT
*G6PC1*	ACTGGCTCAACCTCGTCTTT	CTTTATCAGGGGCACGGAAGT
*SERPINB9*	GGCCCAGGCACTGTCTTTA	ATTGAGCTACCCGGCAACAA
*SLC6A12*	CCTTTCAGGAAGCGTCTGC	GCCACACCCTACAAATGGGT
*TSKU*	GAGACCTTCGGCCTTTTCGAC	CGCCAACACCGACTCATTC
*ZNF165*	ATCCATGGGCAGGACACTTG	CATGGGCTTGAACCTGGAGT
*ANGPTL8*	GCCTGTTGGAGACTCAGATGG	GTGAGGGCCCATAGGATGTG
*CYP24A1*	GCTTACGCCGAGTGTACCAT	GCATGAGCACTGTTCCTTTGG
*ANKRD1*	GCGCCCGAGATAAGTTGCTC	AGTCTCACCGCATCATGCAA
*CYR61*	CTCGCCTTAGTCGTCACCC	CGCCGAAGTTGCATTCCAG

### VDRA and VDRB1 elicit distinct transcriptional programs in HepG2 Cells

**Fig. 2:**
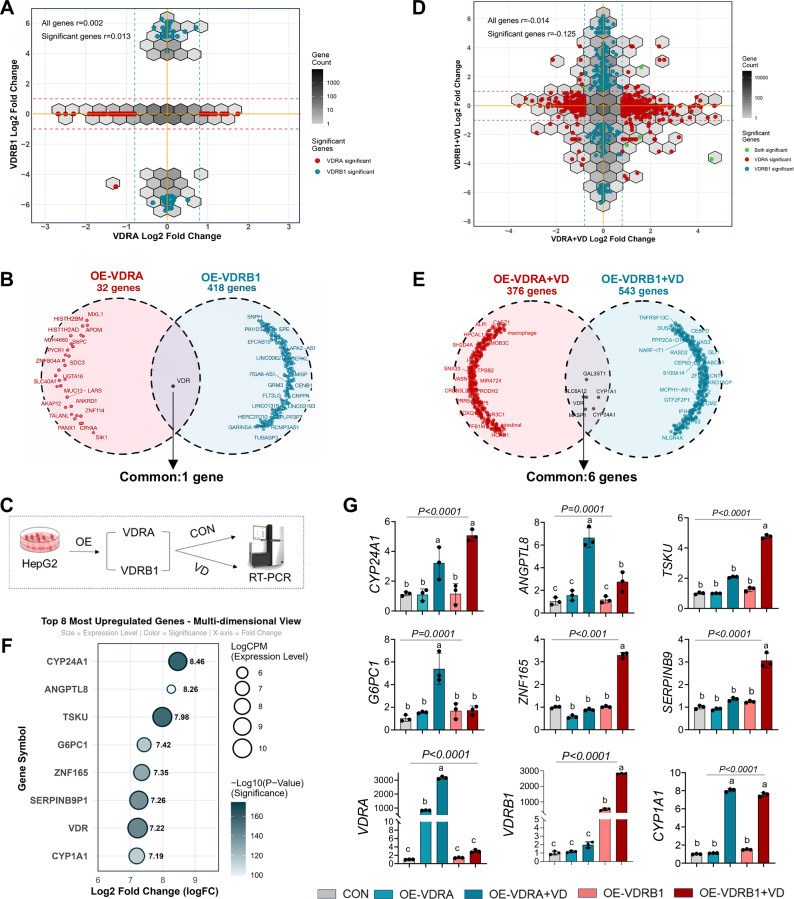
VDRA and VDRB1 regulate distinct target genes in HepG2 cells. **A** Hexbin plot of log2 fold changes in OE-VDRA (x-axis) and OE-VDRB1 (y-axis); **B** Venn diagram of differentially expressed genes (DEGs) in OE-VDRA (32 genes) and OE-VDRB1 (106 genes); **C** Graphical demonstration of RNA-seq and RT-qPCR experiment; **D** A Hexbin plot of log2 fold changes in OE-VDRA + VD (x-axis) and OE-VDRB1 + VD (y-axis); **E** Venn diagram of DEGs in OE-VDRA + VD (376 genes) and OE-VDRB1 + VD (593 genes); **F** Bubble plot shows the top 8 genes upregulated by 1,25(OH)_2_D_3_ in HepG2 cells; **G** RT-qPCR was conducted to detect gene expression triggered by 1,25(OH)_2_D_3_, as illustrated in (**D**) and (**E**); All mRNA expressions were normalized to that of *β-actin*. a, b, and c were used for statistical significance after ANOVA between multiple groups, followed by comparison of the differences between two using the SNK method, a > b > c, ab indicated no difference compared with a, no difference compared with b, a difference compared with c, and greater than c, and so on. +VD, Upon 1,25(OH)_2_D_3_ treatment; OE, Overexpression.

To examine whether VDRA and VDRB1 drive distinct transcriptional programs in HepG2 cells, we overexpressed either VDRA or VDRB1 and performed transcriptome sequencing. Hexbin correlation analysis revealed a weak correlation between the gene expression profiles of VDRA- and VDRB1-overexpressing cells (all genes: r = 0.002; significantly altered genes: r = 0.013) (Fig. 2A). Venn diagram analysis further showed that VDRA overexpression specifically regulated 32 genes, while VDRB1 uniquely regulated 106 genes, with only one overlapping gene (Fig. 2B), indicating distinct transcriptional signatures driven by the two isoforms.

To further determine whether these differences persist upon 1,25(OH)₂D₃ stimulation, HepG2 cells overexpressing VDRA or VDRB1 were treated with 1,25(OH)₂D₃, followed by transcriptome and RT-qPCR analyses (Fig. 2C). Consistent with the above findings, hexbin correlation analysis again demonstrated a weak correlation between VDRA- and VDRB1-mediated transcriptional responses (all genes: r = –0.014; significantly altered genes: r = –0.125) (Fig. 2D). Moreover, Venn analysis identified 376 genes specifically regulated by VDRA and 543 genes uniquely regulated by VDRB1, with only six overlapping genes (Fig. 2E). Together, these results demonstrate that VDRA and VDRB1 exhibit distinct and nonredundant transcriptional regulatory profiles, both under basal and 1,25(OH)₂D₃-stimulated conditions.

RT-qRCR was employed to validate the top 8 up-regulated genes resulting from treatment with 1,25(OH)_2_D_3_ as depicted in Fig. 2F. The data disclosed that HepG2 cells treated with 1,25(OH)_2_D_3_ showed an increase in the expression of *ANGPTL8* and *G6PC1* when subjected to VDRA overexpression, but not with VDRB1 overexpression. Nonetheless, the levels of *SERPINB9, ZNF165* and *TSKU* were conspicuously elevated within 1,25(OH)_2_D_3_-treated VDRB1 overexpression cells, but failed to yield a comparable expression in VDRA overexpressed cells. Both *CYP1A1* and *CYP24A1* were regulated by the overexpression of VDRA and VDRB1 following administration of 1,25(OH)_2_D_3_ (Fig. 2F). In conclusion, the findings corroborated that the binding of 1,25(OH)_2_D_3_ to VDRA and VDRB1 regulates the expression of distinct genes in HepG2 cells.

### VDRA selectively inhibits the YAP transcriptional program independent of VDRB1

**Fig. 3:**
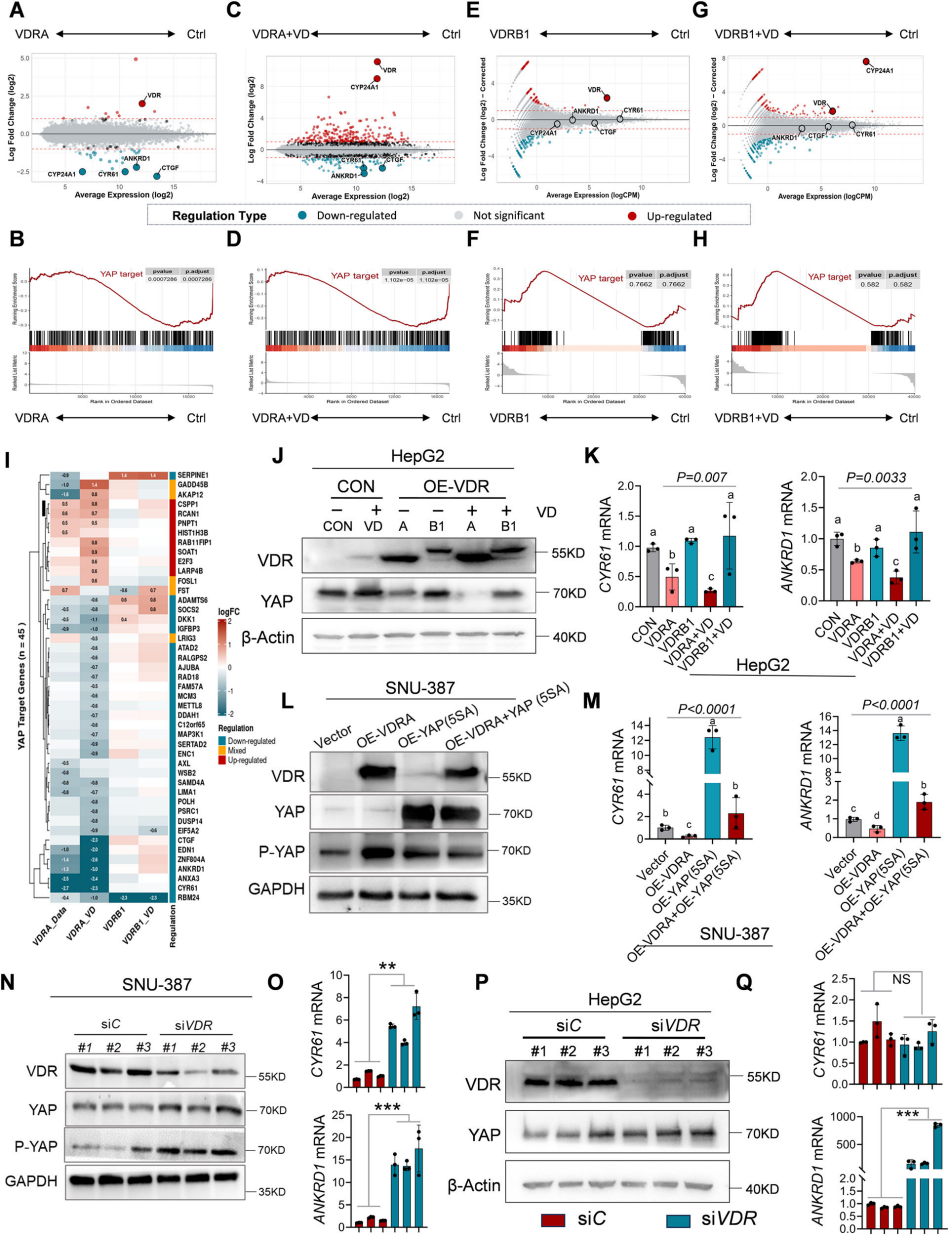
VDRA, rather than VDRB1 suppressed YAP activation. **A**–**G** MA plots of gene expression in VDRA and VDRB1 overexpression with or without 1,25(OH)₂D₃ treatment, key genes are labeled; **B**–**H** GSEA for YAP target genes in VDRA and VDRB1 overexpression with or without 1,25(OH)₂D₃ treatment. The plots show the enrichment score (ES) for YAP target genes, with the x-axis indicating the rank of genes based on their differential expression, and the y-axis representing the normalized enrichment score (NES); **I** Heatmap showing the expression of YAP target genes in VDRA and VDRB1 overexpression with or without 1,25(OH)₂D₃ treatment. Rows represent individual YAP target genes, and columns correspond to each experimental condition; **J**, **N**, **P** YAP and p-YAP expression after overexpression or knockdown of VDR as conformed by western blot; **L** Western blot analysis was performed to investigate the protein levels of VDR, YAP, and phosphorylated YAP (P-YAP) in SNU-387 cells following the overexpression of VDR (OE-VDRA) and/or constitutively active YAP (OE-YAP(5SA)); **K**, **M**, **O**, **Q** RT-qPCR was used to determine the expression of YAP target genes; Subsequent statistical analysis was performed with unpaired two-sided student t-tests or one-way ANOVA with Tukey’s method for multiple comparisons. NS present not statistically significant; *p < 0.05, **p < 0.001, ***p < 0.0001. VD, 1,25(OH)_2_D_3_.

Subsequently, RNA sequencing unveiled that overexpression of VDRA downregulated the expression of YAP target genes in HepG2 cells. Intriguingly, the most notably downregulated gene was *CYR61*, a direct target of YAP, followed by *ANKRD1*, which ranked fifth in the list (Fig. 3A). GSEA enrichment further confirmed that VDRA overexpression robustly curtailed the YAP target gene set (Fig. 3B). Treatment with 1,25(OH)₂D₃ in VDRA-overexpressing HepG2 cells led to a substantial suppression of the top 3 downregulated genes—*ANKRD1*, *CYR61*, and *CTGF*—all of which are established YAP target genes. The GSEA analysis highlighted that 1,25(OH)₂D₃ treatment in VDRA-overexpressing cells exacerbated the repression of the YAP target gene set (Fig. 3C, D). In contrast, overexpression of VDRB1, with or without 1,25(OH)_2_D_3_ treatment, had no discernible impact on the expression of YAP target genes (Fig. 3E–H). Heatmap analysis showed that VDRA overexpression markedly downregulated multiple YAP target genes, with the repression further strengthened upon 1,25(OH)₂D₃ treatment (Fig. 3I). These findings collectively substantiated the notion that VDRA activation exerted a repressive influence on YAP target genes expression within HepG2 cells, while VDRB1 shows no comparable effect.

We next investigated the regulatory impact of VDR on YAP signaling activity. In HepG2 cells, ectopic expression of VDRA led to a conspicuous reduction of total YAP protein, which was further reduced following 1,25(OH)₂D₃ treatment (Fig. 3J). Consistent with the reduction in YAP protein, the transcriptional activity of YAP was also markedly inhibited, as evidenced by the downregulated mRNA levels of its downstream target genes, *CTGF* and *CYR61*, particularly upon 1,25(OH)₂D₃ treatment (Fig. 3K). This reduction was also evident at the mRNA level, as the expression of YAP target genes *CTGF* and *CYR61* was notably suppressed in VDRA-overexpressing cells, with an even stronger effect observed upon 1,25(OH)₂D₃ treatment (Fig. 3K). Intriguingly, VDRB1 overexpression failed to modulate YAP or its downstream targets regardless of 1,25(OH)₂D₃ stimulation, highlighting the functional specificity of the VDRA isoform (Fig. 3J, K).

To validate these findings in a more physiologically relevant context, we employed SNU-387 cells, which harbor high endogenous VDR expression (Fig. 1J). Consistent with our observations in HepG2 cells, VDRA overexpression in SNU-387 cells significantly augmented YAP phosphorylation at Ser127 (p-YAP) while concomitantly suppressing the expression of its downstream targets (Fig. 3L, M). Crucially, this VDRA-mediated repression of YAP signaling was substantially abrogated by the introduction of the constitutively active YAP-5SA mutant (Fig. 3L, M). Furthermore, knockdown of endogenous VDR in SNU-387 cells led to a marked decrease in p-YAP levels and a concomitant increase in total YAP and its targets, *CYR61* and *ANKRD1* (Fig. 3N, O). This regulatory pattern was faithfully recapitulated in HepG2 cells, where VDR knockdown effectively upregulated both YAP and its target genes (Fig. 3P, Q). These findings complemented the RNA sequencing data, suggesting that VDRA discernibly suppresses YAP activation in HepG2 and SNU-387 cells.

### Activated VDRA suppresses HCC proliferation by restricting YAP nuclear translocation

**Fig. 4:**
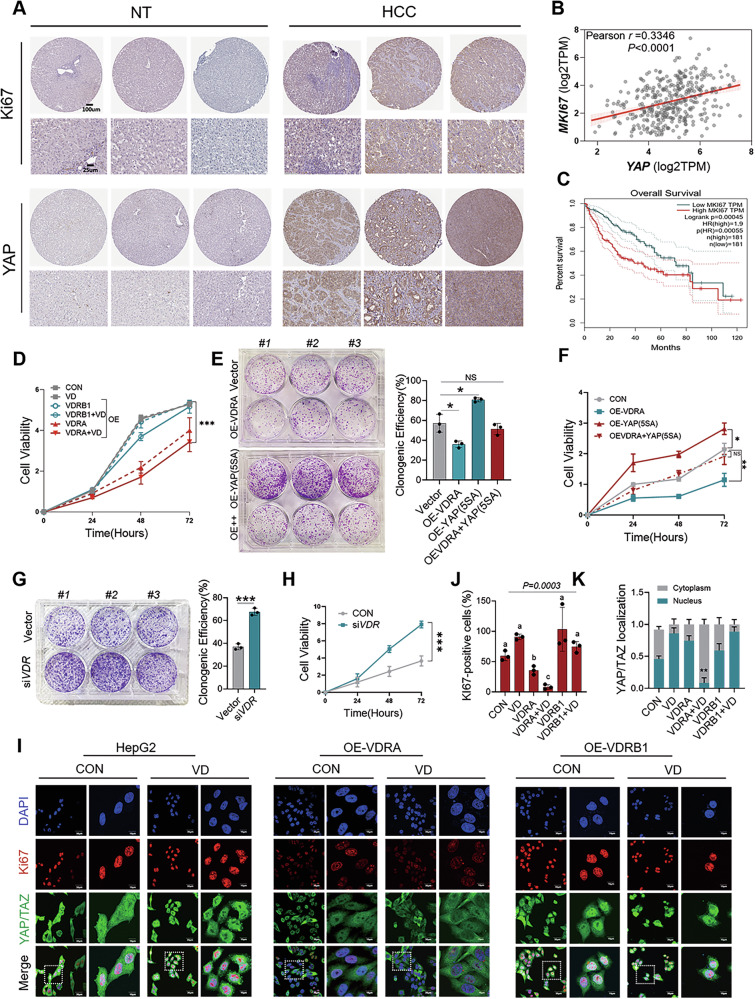
VDRA sensitivity to 1,25(OH)_2_D_3_ inhibits HepG2 proliferation by suppressing YAP nuclear translocation. **A** Immunohistochemical results of YAP and Ki67 in tissues from 3 normal livers and 3 HCC samples. **B** Correlation analysis of Ki67 with YAP expression in 366 HCC samples (TCGA). **C** Overall survival analysis of Ki67 in hepatocellular carcinoma (TCGA). **D** CCK-8 was performed for HepG2 proliferation; **E**, **G** The proliferative ability of SNU-387 cells was evaluated by colony formation assays, and representative images and quantitative analyses are shown; **F**, **H** Cell proliferation of SNU-387 cells was assessed using the CCK-8 assay at the indicated time points; **I** YAP and Ki67 expression in HepG2 cells as confirmed by Immunofluorescence. **J** Quantification of YAP expression in the nucleus and cytoplasm. **K** The number of Ki67-positive cells. All mRNA expressions were normalized by *β-actin*; a, b, and c were used for statistical significance after ANOVA between multiple groups, followed by comparison of the differences between two using the SNK method, a > b > c, ab indicated no difference compared with a, no difference compared with b, a difference compared with c, and greater than c. OE, Overexpression. Pearson correlation was used for correlation analysis, and *p* < 0.05 was considered statistically significant.

Immunohistochemical array showed YAP and cancer proliferation marker, *Ki67*, displayed minimal expression in normal liver tissue but exhibited significant upregulation in HCC specimens (Fig. 4A, Table S1–S3). Conversely, 1,25(OH)_2_D_3_ activating enzyme markedly elevated expression in healthy liver tissues, with a substantial decline observed in HCC tissues (Fig. S2, Table S4-S6). The bioinformatics analysis further corroborated these findings, within HCC tissues, *Ki67* expression positively correlated with *YAP* (Fig. 4B), and negatively correlated with 1,25(OH)_2_D_3_ activating enzyme (Fig. S2C). Moreover, a negative correlation emerged between the expression of 1,25(OH)_2_D_3_ activating enzyme and *YAP* (Fig. S2B). Additionally, abundant levels of *Ki67* wherein livers of patients suffering from HCC predict transient survival time (Fig. 4C). Overall, YAP expression, unlike that of the 1,25(OH)₂D₃-activating enzyme, was negatively correlated with the proliferative capacity of HCC tissues.

We sought to investigate whether VDR activation exerts anti-proliferative effects in HCC. CCK-8 assays revealed that overexpression of VDRA, with or without 1,25(OH)₂D₃ treatment, effectively inhibited HepG2 cell proliferation. However, this inhibitory effect was notably absent in VDRB1-overexpressing cells (Fig. 4D). Similarly, in SNU-387 cells, VDRA overexpression significantly suppressed cell proliferation and colony-forming capacity (Fig. 4E, F). To confirm whether this growth inhibition was mediated by YAP, we performed a genetic rescue assay. The results showed that the anti-proliferative and anti-colony effects induced by VDRA were substantially abrogated by the overexpression of YAP-5SA (Fig. 4E, F). Conversely, knockdown of VDR in SNU-387 cells markedly promoted cell proliferation (Fig. 4G, H). Immunofluorescence staining disclosed no discernible variances in the levels of YAP and Ki67 expression between the HepG2 cells treated with and without 1,25(OH)_2_D_3_ (Fig. 4I, J). Notably, activation of VDRA attenuated the YAP fluorescence signal and the number of Ki67-positive cells. While it did not alter the nuclear translocation of YAP (Fig. 4I, K). Importantly, the overexpression of VDRA cells led to a notable decrease in the number of Ki67-positive cells and facilitated the translocation of YAP into the cytoplasm upon treatment with 1,25(OH)_2_D_3_ (Fig. 4I–K). There was no discernible effect on the expression of YAP and Ki67 in HepG2 cells that either overexpressed VDRB1 or were treated with 1,25(OH)_2_D_3_, as shown in Fig. 4I–K. In summary, our findings robustly demonstrated that the sensitivity of VDRA to 1,25(OH)_2_D_3_ suppressed YAP transcription, consequently inhibiting the proliferation of HepG2 cells.

### VDRA activation attenuates tumorigenicity in human liver cancer organoids

**Fig. 5:**
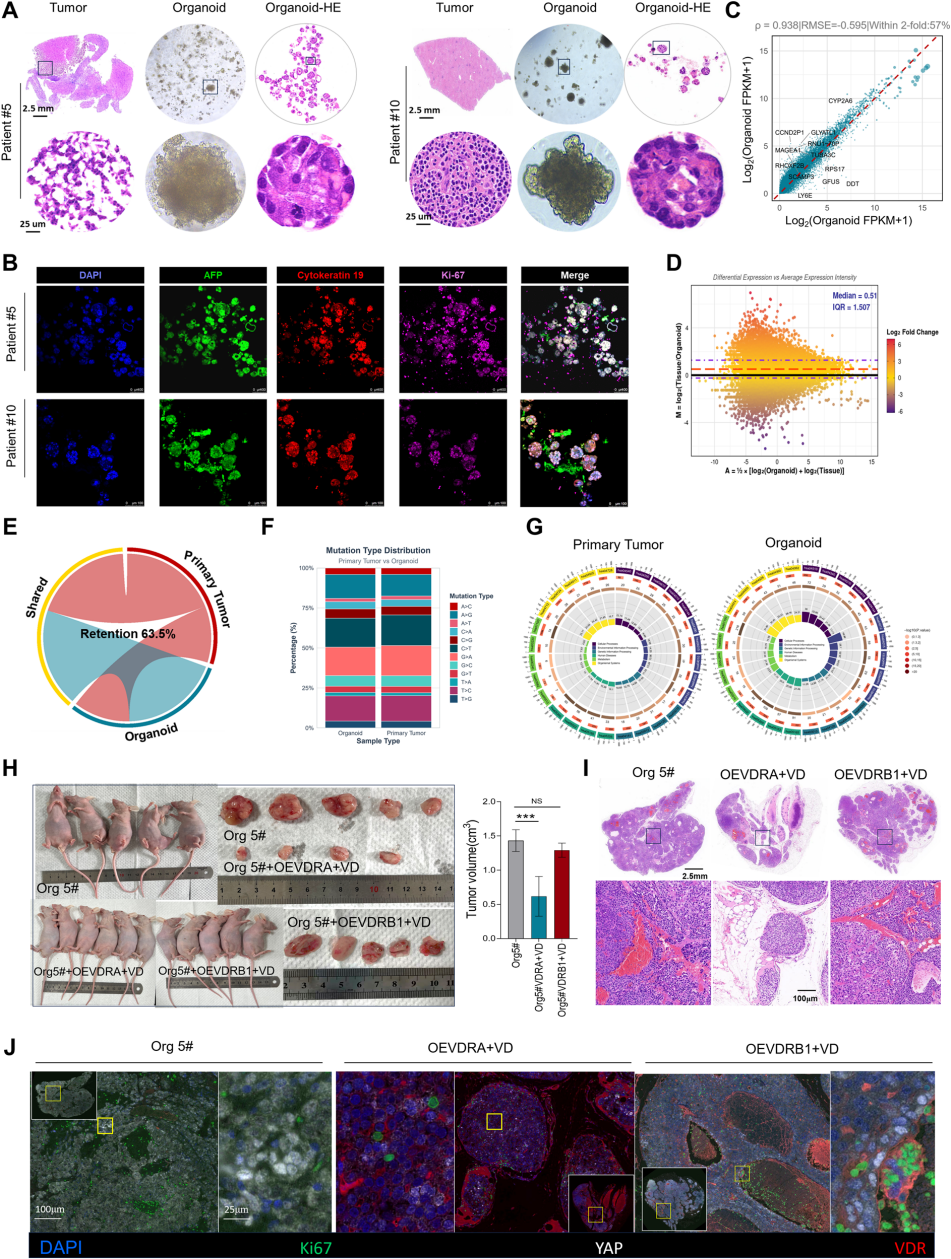
VDRA but not VDRB1 activation attenuates tumorigenicity in human liver cancer organoids. **A** Morphological features and H&E staining of human HCC tumor tissues and their corresponding cultured organoids; **B** The immunofluorescence staining shows the expression of liver cancer markers in the organoids; **C** Transcriptomic profiling and correlation analysis of gene expression between human HCC tumor tissues and their corresponding organoid cultures, revealing a strong positive correlation; **D** MA plot showing the differential gene expression between human HCC tumor tissues and their corresponding organoid cultures; **E** The Venn diagram shows the proportion of mutations shared between tumor tissues and organoids, with 63.5% of mutations retained in the organoid model; **F** Mutation type distribution between primary tumor tissues and organoid cultures; **G** Enrichment analysis circular plots illustrating the impact of mutated genes on function in human HCC primary tumor tissues (left) and organoid cultures (right); **H** Tumor formation following subcutaneous transplantation of organoids in nude mice; **I** Hematoxylin and eosin (H&E) staining of subcutaneous tumor tissues derived from organoids; **J** Multicolor immunofluorescence analysis of subcutaneous tumor tissues formed by organoids.

To further elucidate the impact of VDRA on HCC proliferation, we established organoid cultures using residual tumor tissues obtained from HCC patients during surgical resection. Importantly, these cultured organoids consistently retained pathological features and markers closely resembling those of the corresponding primary tumors (Fig. 5A–C). Subsequently, we performed transcriptome sequencing and whole-exome sequencing (WES) on both organoids and their corresponding HCC tissues. Transcriptomic profiling demonstrated a strong correlation in gene expression between organoids and primary tumors (ρ = 0.938; Fig. 5C), accompanied by minimal overall expression variability (IQR = 1; Fig. 5D). WES analysis further revealed that 63.5% of the somatic mutations identified in primary tumors were retained in the matched organoids (Figs. 5E, S3A, B). In addition, organoids and primary tumors exhibited a highly concordant mutational spectrum (Fig. 5F) as well as similar enrichment patterns of signaling pathways influenced by these mutated genes (Fig. 5G). Collectively, these results indicate that the established organoid model faithfully preserves the genetic landscape of the primary tumors, thereby providing a robust and reliable platform for subsequent functional investigations.

In established HCC organoid models, we ectopically expressed VDRA or VDRB1 (Figure. S3C). Following three days of 1,25(OH)₂D₃ treatment, the organoids were transplanted subcutaneously into nude mice. Tumorigenicity assays revealed that VDRA overexpression markedly reduced tumor volume compared with controls, whereas VDRB1 overexpression showed no significant effect (Fig. 5H). H&E staining demonstrated that VDRA overexpression led to increased tumor necrosis and reduced cellular proliferation, reflecting diminished intratumoral heterogeneity, whereas VDRB1 overexpression resulted in no significant histological changes relative to controls (Fig. 5I). Consistently, immunohistochemical analyses indicated that VDRA overexpression suppressed Ki67 and YAP expression within tumor tissues (Fig. 5J). Collectively, these findings indicate that VDRA activation restrains HCC organoid proliferation and attenuates YAP expression.

### VDRA but not VDRB1 efficiently translocate to the nucleus dependent on 1,25(OH)₂D₃

**Fig. 6:**
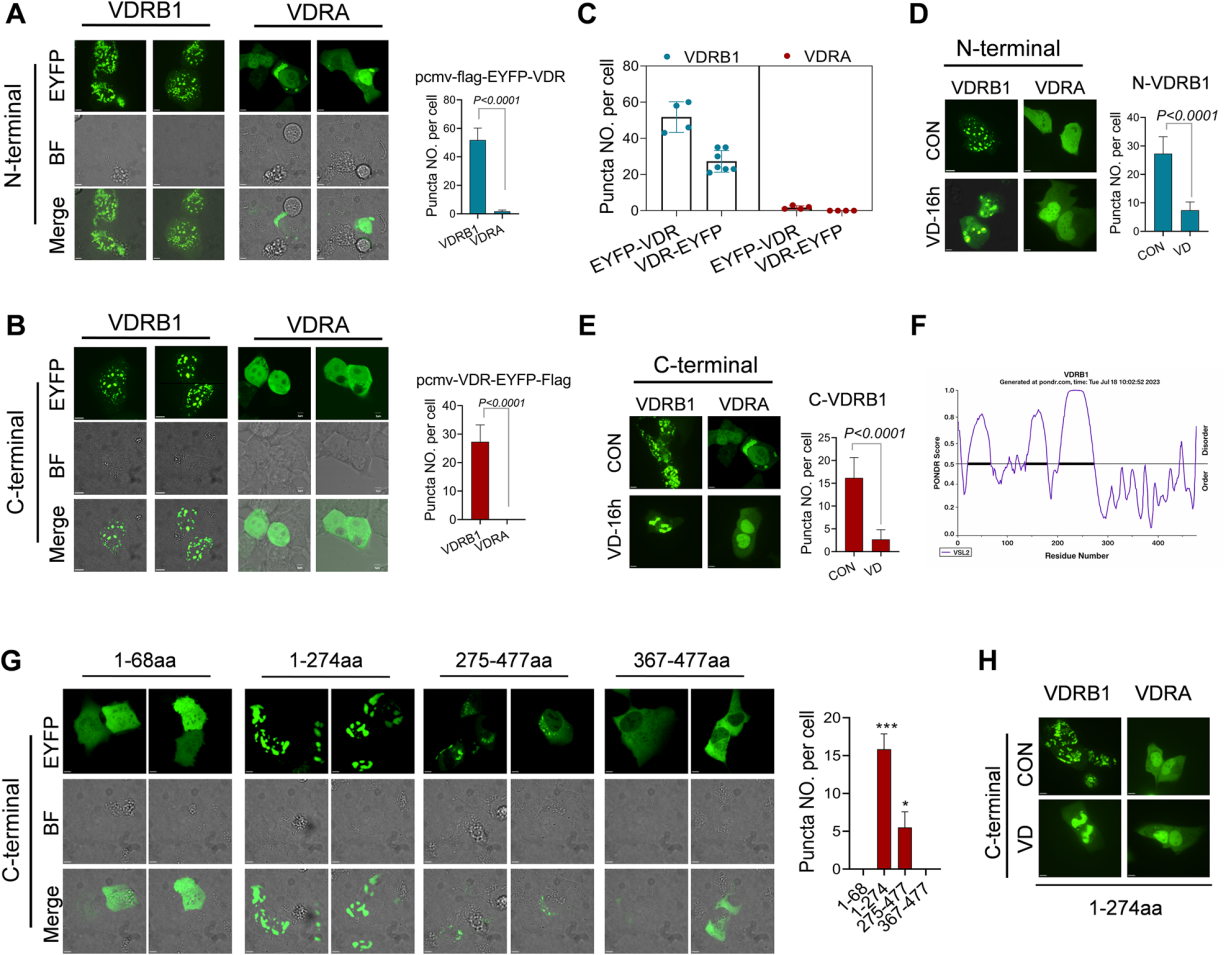
VDRB1 had a puncta expression and did not enter the nucleus after treatment with 1,25(OH)_2_D_3_. **A**, **B** EYFP was fused at N- or C-terminal of VDRA and VDRB1 and were visualized under a microscope; **C** Quantification of puncta formed by VDRA or VDRB1; **D**, **E** 1,25(OH)_2_D_3_ induced VDRA and VDRB1 proteins were visualized under a microscope; **F** PONDR predicts IDRs of VDRB1; **G** Based on the PONDR prediction, four truncated forms of VDRB1 were constructed and fused with EYFP; **H** 1-274aa of VDRA and VDRB1 protein fusions with EYFP treated with or without 1,25(OH)_2_D_3_, and was visualized under a microscope. Analysis of differences between the two groups was performed with unpaired two-sided Student t-tests, and *p* < 0.05 was considered statistically significant.

To investigate the mechanism underlying VDRB1’s inability to suppress YAP, we generated N- and C-terminal EYFP fusion constructs for both VDRA and VDRB1. Confocal imaging revealed that VDRB1 consistently formed distinct cytoplasmic puncta regardless of the EYFP tag position, whereas VDRA exhibited a predominantly diffuse distribution (Fig. 6A, B). Interestingly, N-terminally tagged VDRB1 (EYFP-VDRB1) displayed a higher density of puncta compared to the C-terminally tagged version (VDRB1-EYFP) (Fig. 6C).

Upon treatment with 1,25(OH)₂D₃, the cytoplasmic puncta of both VDRB1 constructs coalesced into larger aggregates (Fig. 6D, E). Both EYFP-VDRA and VDRA-EYFP underwent robust nuclear translocation following ligand stimulation (Fig. 6D, E). Bioinformatic analysis using PONDR predicted that the N-terminal 1–274 amino acid (aa) region of VDRB1 contains a significant intrinsically disordered region (IDR) (Fig. 6F), which is often associated with protein clustering. To identify the specific domain responsible for this punctate distribution, we generated four VDRB1 truncation mutants (1–68 aa, 1–274 aa, 175–477 aa, and 267–477 aa). Fluorescence imaging demonstrated that the 1–274 aa fragment was sufficient to recapitulate the punctate expression pattern observed in full-length VDRB1 (Fig. 6G). Furthermore, while the VDRB1 (1–274 aa) truncated formed enlarged aggregates upon 1,25(OH)₂D₃ treatment, the corresponding VDRA (1–274 aa) fragment successfully translocated to the nucleus (Fig. 6H).

**Fig. 7:**
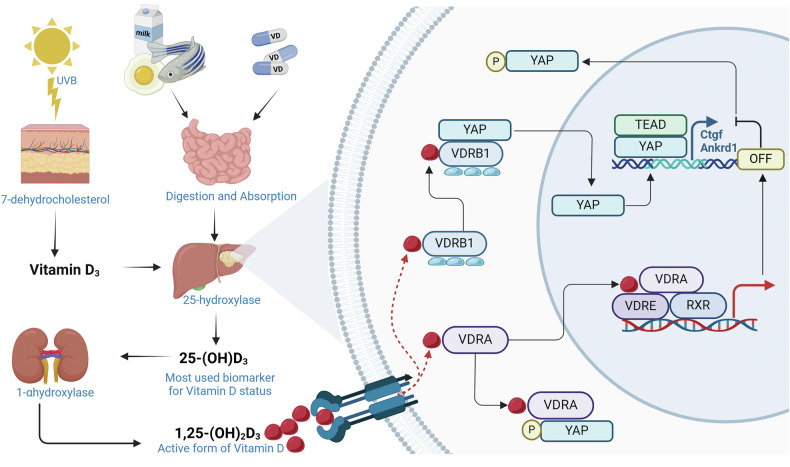
Synthesis of active vitamin D in vivo and its mechanism of action in liver cancer cells. Ultraviolet rays from the sun induce the synthesis of the vitamin D precursor (Vitamin D_3_) in the skin, while dietary vitamin D is absorbed through the intestine. Both forms are transported to the liver and kidneys, where they undergo two activation steps to produce the biologically active form, 1,25(OH)₂D₃. The active 1,25(OH)₂D₃ then enters target cells, such as liver cancer cells, and binds to its receptors, VDRA and VDRB1. Activated VDRA translocated to the nucleus and exerts its transcriptional function to inhibit YAP activity, thereby suppressing liver cancer proliferation. In contrast, activated VDRB1 forms a phase-separated structure and does not inhibit YAP activity, thus having no effect on liver cancer proliferation.

Given that VDR must enter the nucleus to exert its function as a transcription factor, our data suggest that 1,25(OH)₂D₃ promotes VDRA nuclear entry while triggering the cytoplasmic sequestration of VDRB1 within punctate structures. This spatial restriction likely prevents VDRB1 from accessing the nucleus, thereby precluding its regulation of YAP-mediated transcription (Fig. 7).

## Discussion

The eight transcripts of the VDR exhibited low expression levels in parenchymal liver cells. Our study revealed that both VDRA and VDRB1 transcripts were upregulated in response to 1,25(OH)2D3 in HCC. Remarkably, ectopic VDRA notably augmented the anti-proliferative effect of 1,25(OH)_2_D_3_ on HepG2 cells by robustly curtailing YAP’s activity. VDRB1 was unable to perform this function, as it mechanically exhibited puncta expression, resulting in the inaccessibility to enter the nucleus induced by 1,25(OH)_2_D_3_. Additionally, the findings from the analysis of human HCC tissue array uncovered a negative correlation between the expression of CYP27A1, which encodes 25-hydroxyvitamin D3 synthase in the liver, and the levels of YAP. In addition, CYP27A1 has been found to be inversely correlated with the proliferative potential of HCC.

The present study demonstrated the presence of VDR in almost all human organs, with a low expression in the liver. The ubiquitous distribution of the VDR suggests that the physiological functions of vitamin D-VDR extend beyond the regulation of calcium homeostasis [38]. Although VDR expression levels were relatively minimal in liver tissue, they were conspicuously elevated in Kupffer and HSC cells [39], which indicates that VDR has a remarkable role in the liver [39]. The primary variant of the VDR is VDRA, which consists of 427 amino acids with a molecular weight of 48 kDa. Another isoform, VDRB1, exhibits an elongation of approximately 50 amino acids in its N-terminal domain (477 amino acids, 54 kDa) due to the initiation site (ATG) located in exon 1 d (in contrast to VDRA, which has a unique start site in exon). In human kidney, intestine, and kidney epithelial cell lines, VDRB1 has been identified [34, 40]. This extension enables distinct ligands (calcitriol or lithocholic acid) to elicit specific responses in various tissue types, indicating that the activation of VDRA and VDRB1 is specific to both the ligand and the tissue involved [35, 41, 42]. In organs with low VDR expression, such as the liver, VDRA has a comparable ligand-dependent transcriptional ability to VDRB1. The consistency of our findings is supported by the presence of VDRA and VDRB1, homologous isoforms of VDR, in human HCC tissues, where they are involved in the regulation of distinct target genes. Thereby, variations in transcription patterns and strengths of the VDR can occur due to its isoforms, VDRA and VDRB1.

YAP plays a crucial role as a component of the hippo pathway, which serves as a significant driver of HCC proliferation [12, 13]. Besides, YAP activation was an independent predictor of HCC prognosis and was associated with more aggressive types of HCC [43, 44]. Given the potential oncogenic properties of YAP, a considerable number of researchers have demonstrated significant interest in developing small-molecule inhibitors targeting YAP as potential therapeutic agents to suppress the proliferation of HCC. It is noteworthy that the vitamin D-VDR exhibits contrasting regulatory effects on YAP in various diseases. Some studies indicate that 1,25(OH)_2_D_3_/VDR promotes YAP activation in response to high levels of bile acids accumulated in metastatic lymph nodes [45], as well as fibrogenesis in conditions such as cholestasis [46] or bile duct ligation [47]. Conversely, another work disclosed that VDR inhibits tumor proliferation and migration by suppressing YAP activity [48]. In line with our research findings, we observed that VDRA, but not VDRB1, down-regulates YAP activation in HepG2 cells. The observed variances may originate from the diverse functions of YAP in specific developmental or pathological processes. This statement elucidates the distinct functions of the VDR in the hippo pathway signaling and provides a rationale for the varying effects of vitamin D in different diseases [49].

In the present study, we observed that VDRB1 possesses a distinct tendency to form punctate structures within the cytoplasm. Unlike VDRA, VDRB1 fails to undergo nuclear translocation even upon stimulation by 1,25(OH)₂D₃. Previous literature has established that intrinsically disordered regions (IDRs) in transcription factors can facilitate protein clustering or focal aggregation, which often results in impaired nuclear import and a subsequent decline in transcriptional output [50, 51]. Our bioinformatic analysis consistently identified a significant IDR spanning residues 1–274 of VDRB1, which likely serves as the structural basis for this cytoplasmic sequestration. Recent studies have underscored that the spatial organization and condensation of regulatory proteins play a pivotal role in modulating the oncogenic activity of YAP, and disrupting such localized accumulation has been shown to impede tumorigenesis [52]. While our findings suggest that the functional divergence of VDRB1 may stem from its physical sequestration within cytoplasmic puncta, the precise biophysical nature of these structures—specifically whether they represent liquid-liquid phase separation (LLPS)—remains to be definitively characterized. Further investigation, including in vitro reconstitution assays with purified protein and cellular tests such as FRAP or 1,6-hexanediol sensitivity, is warranted to confirm the LLPS hypothesis and determine if these puncta directly recruit YAP.

Despite the conceptual advance provided by this study, several limitations should be noted. First, the broader molecular landscape of VDRB1-mediated sequestration remains partially obscured; future studies employing high-resolution mass spectrometry are necessary to map the VDRB1 interactome and identify whether other Hippo pathway components are co-sequestered. Second, while VDRA clearly modulates YAP activity, the exact molecular interface—whether through direct physical binding or indirect signaling crosstalk—requires further structural elucidation. Finally, although our patient-derived organoid (PDO) model provides a physiologically relevant platform, our reliance on a single-patient cohort is a recognized limitation. Given the profound genetic and phenotypic heterogeneity of HCC, validating these findings in larger, multi-patient PDO-based xenograft models will be essential to substantiate the translational potential of isoform-specific VDR-targeted therapies across diverse clinical contexts.

In conclusion, our study demonstrates that VDRA, but not VDRB1, significantly enhances the anti-proliferative effects of 1,25(OH)_2_D_3_ by robustly inhibiting YAP activation in HCC. While VDRB1 remains functionally inert in this context and undergoes cytoplasmic sequestration into puncta, VDRA emerges as a primary mediator of tumor suppression. However, the spatiotemporal complexity of VDR signaling—potentially modulated by the VDRA/VDRB1 ratio—suggests that its therapeutic application must be tailored to specific oncogenic contexts. While further in vivo studies are necessary to fully elucidate the role of VDRA in diverse liver pathologies, this study identifies VDRA as a promising and actionable target for inhibiting YAP-driven proliferation in HCC.

## Methods

### Data sources

VDR expression profiles across diverse human tissues were retrieved from the GTEx database (https://gtexportal.org/). RNA-seq data and comprehensive clinical metadata for Hepatocellular Carcinoma (HCC) patients, as well as other tumor types, were accessed via the TCGA data portal (https://www.cancer.gov/tcga). Raw transcriptomic expression data for VDR were obtained from TSVdb (http://www.tsvdb.com/), while VDR expression levels in HCC cell lines were sourced from the GSE36133 dataset within the Gene Expression Omnibus (GEO) database. Additionally, immunohistochemistry (IHC) data were provided by The Human Protein Atlas (THPA) (https://www.proteinatlas.org/). Crucially, all available samples from these designated datasets were included in the analysis, and no samples were excluded based on any prior criteria.

### Transcriptome Sequencing and Bioinformatic Analysis

Transcriptome sequencing (RNA-seq) was performed by Azenta Life Sciences (formerly GENEWIZ; Suzhou, China). High-quality reads were aligned to the reference genome, and the identification of differentially expressed mRNAs (DEGs) was conducted using the edgeR package within the R environment. Significant DEGs were defined based on the criteria of an absolute log2-fold change ( | log2FC | ) > 1 and a Benjamini-Hochberg adjusted *p*-value < 0.05. Visualization of the transcriptomic distribution was implemented via volcano plots using the ggplot2 package. Additionally, Gene Set Enrichment Analysis (GSEA) was performed using the GSEA desktop application (v4.0.3; Broad Institute) to identify significantly enriched biological pathways and functional signatures.

### Establishment and culture of patient-derived hepatocellular carcinoma organoids

Fresh HCC tissue samples (~1 cm³) were obtained intraoperatively from patients undergoing surgical resection at Fujian Provincial Hospital. All procedures were conducted in accordance with the Declaration of Helsinki and approved by the Ethics Committee of Biomedical Research, Fujian Medical University (Approval No. FUYI-ETHICS-2025-57). Written informed consent was obtained from all patients prior to tissue collection.

Tumor specimens were immediately transferred to the laboratory in a tissue preservation solution on ice. The samples were minced into small fragments and enzymatically digested to obtain single-cell suspensions. Cells were subsequently embedded in Matrigel domes and cultured using the Hepatocellular Carcinoma Organoid Kit (Serum-free, K2105-HCC; BioGenous, China) according to the manufacturer’s instructions. The culture medium was refreshed every 2–3 days, and organoids were passaged every 7–10 days by mechanical disruption and re-embedding in fresh Matrigel.

### Lentiviral vector construction and cloning

The lentiviral vectors expressing VDRA and VDRB1 were constructed using EcoRI and BamHI restriction enzymes to ligate the amplified gene fragments into either the pCMV-flag-EYFP vector or the pCMV-EYFP-Flag lentiviral expression vector (provided by Mr. Caiyong Ye). VDRA and VDRB1 were amplified by PCR from HepG2 cells using Phusion high-fidelity DNA polymerase (NEB, Ipswich, MA, USA). The *VDRA* and *VDRB1* genes were amplified with the following primers: VDRA F: 5′-GGAATTCCACCATGGAGGCAATGGCGGCCA-3′; VDRB1 F: 5′-GGAATTCCACCATGGAGTGGAGGAATAAG-3′；Common reverse primer: 5′-CGGGATCCGGAGATCTCATTGCCAAAC-3′.

The PCR products were digested with EcoRI/BamHI and ligated into the lentiviral vectors, yielding the following constructs: pCMV-VDRA-EYFP, pCMV-VDRB1-EYFP, pCMV-EYFP-VDRA, and pCMV-EYFP-VDRB1. For the construction of truncated VDRB1 variants, the pCMV-VDRB1-EYFP plasmid was used as a template. The following primers were used to generate the truncations:

**Table:** 

**Names**	**Primer**
VDRB1(1-68aa) Forward	5′-GGAATTCCACCATGTCCATGCTGCCCCACCTG-3′
VDRB1(1-68aa) Reverse	5′-CGGGATCCCGGTCAAGTCTCCAG-3′
VDRB1(1-274aa) Forward	5′-GGAATTCCACCATGTCCATGC-3′
VDRB1(1-274aa) Reverse	5′-CGGGATCCGAGCTGGGACAGCTCTAG-3′
VDRB1(257-477aa) Forward	5′-GGAATTCCACCATGTCCATGCTGCCCCACCTG-3′
VDRB1(367-477aa) Forward	5′-GGAATTCCACCATGGTGGGACTGAAGAAGCTG-3′
VDRB1(257-477aa) and VDRB1(367-477aa) Reverse	5′-GGAATTCTTACTTGTCGTCATCGTC-3′

The truncated VDRB1 sequences were then inserted into the pCMV-flag-EYFP vector following the same cloning procedure.

### Transfected cells

HepG2 and SNU-387 cell lines were purchased from Procell Life Science & Technology (Wuhan, China) and SUNNCELL (Wuhan, China), respectively. Both lines were authenticated by STR profiling and confirmed to be mycoplasma-free. For transfection, cells were seeded in 6-well plates to reach 60–70% confluence. Plasmid DNA was transfected using Lipofectamine 3000 (Thermo Fisher Scientific) in Opti-MEM medium, following the manufacturer’s instructions. The culture medium was refreshed 12 h post-transfection, and cells were harvested after 24 h for further analysis of transfection efficiency at both RNA and protein levels.

### Construction of HepG2 cell lines and HCC organoids stably overexpressing VDRA and VDRB1

The full-length coding sequences of VDRA and VDRB1 were cloned into the FUW-tetO-IRES-puro vector, which was kindly provided by Mr. Caiyong Ye. Viral particles were generated in 293 T cells and subsequently used to transduce HepG2 cells or organoids. For cell transduction, HepG2 cells were directly infected with the viral supernatant and selected with puromycin to establish stable lines overexpressing either VDRA or VDRB1. For organoid transduction, cultures were first dissociated into single-cell suspensions prior to viral infection, and stable overexpression was achieved following puromycin selection.

### Tumor xenograft model

To establish the subcutaneous xenograft model, stably overexpressing VDRA or VDRB1 HCC organoids and their respective controls were dissociated into single-cell suspensions and resuspended in an ice-cold PBS/Matrigel mixture (1:1, v/v) at a density of 1 × 10^7^ cells/mL. A 100 μL aliquot of the suspension was subcutaneously injected into the left and right inguinal regions of 4–6-week-old male BALB/c nude mice (*n* = *6* sites per group; Beijing Vital River Laboratory Animal Technology Co., Ltd.). Mice were randomly assigned to experimental groups, and tumor measurements were performed by investigators blinded to the treatment allocations. Tumor growth was monitored every 5 days. When tumor volumes reached the predefined ethical endpoint of 1.5 cm³, mice were euthanized, and tumors were excised for subsequent histological and molecular analyses. All animal procedures were approved by the Institutional Animal Care and Use Committee of Fujian Medical University (IACUC FJMU 2025-Y-0254) and conducted in accordance with institutional and national guidelines for animal welfare.

### siRNA-mediated knockdown and transfection cells

Small interfering RNAs (siRNAs) targeting VDR and a negative control were acquired from RIBOBIO. The cells were transfected with siRNAs using Lipofectamine RNAiMAX (Thermo Fisher Scientific) according to the manufacturer’s instructions. Unless specified otherwise, cell analysis was conducted 48 hours post-transfection. The target sequences are as follows: VDR1#: 5′-CCTCCAGTTCGTGTGAATGAT-3′; VDR2#: 5′-CTCCTGCCTACTCACGATAAA-3′; VDR3#: 5′-TTGGCTTTGCTAAGATGATAC-3′.

### Immunofluorescence staining

HepG2 cells (with or without VDRA/VDRB1 overexpression) were cultured on glass coverslips, fixed with 4% paraformaldehyde for 30 minutes, and blocked with 5% BSA for 1 hour at room temperature. Cells were incubated overnight at 4°C with primary antibodies against Ki67 (CST #9449, 1:200) and YAP (CST #14074, 1:200). Human HCC organoids were sectioned, deparaffinized, rehydrated, and subjected to antigen retrieval. Sections were blocked with 5% BSA for 1 hour and incubated overnight at 4°C with primary antibodies targeting Cytokeratin 19 (Proteintech, 60187-1-Ig), Ki67 (Invitrogen, 14-5698-82), and AFP (Proteintech, AF5369). After primary antibody incubation, both cells and organoid sections were washed and incubated with secondary antibodies for 1 hour at room temperature: Alexa Fluor 647–anti-mouse IgG (Proteintech, RGAM005, 1:200), Cy3–Goat anti-rabbit IgG (Proteintech, SA00009-2, 1:200), and Alexa Fluor™ 488–Goat anti-rat IgG (Invitrogen, A-11006, 1:200). Nuclei were counterstained with DAPI. Images were captured using a Leica TCS SP5 confocal microscope (Leica Microsystems, Wetzlar, Germany).

### RNA extraction and real-time quantitative RT-qPCR

Total RNA was extracted from HepG2 cells following the manufacturer’s instructions using the Fast pure cell/tissue total RNA isolation kit V2 (Vazyme, RC112-01). The Hiscript® III reverse transcriptase kit (Vazyme) was employed to perform reverse transcription on 1 μg of total RNA for cDNA synthesis. RT-PCR analysis was subsequently conducted on the cDNA. The mRNA expression levels were normalized using β-actin as a reference gene. The specific primer sequences are listed below.

### Western blotting analysis

Immunoblotting experiments were conducted following the procedures outlined in prior research. Protein samples were separated on polyacrylamide gels, subsequently transferred to PVDF membranes, blocked using 5% BSA, and then subjected to incubation with suitable primary antibodies. The membrane was subsequently washed and then incubated with secondary antibodies that are specific to the particular species. All signals were detected utilizing an enhanced chemiluminescence (ECL) solution. The study utilized the following antibodies: VDR (CST, #12550), YAP (CST #2947), TAZ (CST, 4883), Phospho-YAP1 (S127) (HUABIO, ET1611-69), β-actin (Proteintech, 66009-1-Ig) and GAPDH (Proteintech, 60004-1-Ig).Western blot secondary antibodies, including anti-mouse IgG (# PR30012) and anti-rabbit IgG (# PR30011), were procured from Proteintech.

### Cell proliferation assays

Short- and long-term cell proliferation were evaluated using CCK-8 and colony formation assays, respectively. Stably transfected HepG2 and SNU-387 cells, treated with or without 100 nM 1,25(OH)_2_D_3_, were either seeded in 96-well plates (5000 cells/well) for CCK-8 analysis at 24–72 h or in 6-well plates (600 cells/well) for a 2-week colony formation assay. For the CCK-8 assay, absorbance at 450 nm was recorded following a 3-h incubation with 10% reagent (MedChemExpress, USA). For colony formation, resulting colonies were fixed with 4% paraformaldehyde, stained with 0.1% crystal violet, and photographed for quantification.

### Statistical analysis

Gene expression profiles and clinical metadata for Hepatocellular Carcinoma (HCC) were retrieved from the Cancer Genome Atlas (TCGA) database. Statistical analyses were performed using SPSS (v23.0; IBM Corp., Armonk, NY, USA) or R software. Quantitative experimental data are presented as mean ± SEM from at least three independent biological replicates (*n* = *3*). For comparisons between two groups, a two-tailed unpaired Student’s t-test was employed. For multi-group comparisons, one-way analysis of variance (ANOVA) was conducted, followed by the Student-Newman-Keuls (SNK) post-hoc test. Statistical significance across multiple groups is denoted by lowercase letters (a, b, c, or d), where *a* > *b* > *c* > *d*; groups sharing the same letter (e.g., “ab”) indicate no significant difference between them, whereas different letters indicate a significant difference (*p* < 0.05). All statistical significance levels were set at *p* < 0.05.

## Supplementary information

Supplementary data

## Data Availability

The data and materials supporting the findings of this study are available from the corresponding authors upon reasonable request. The raw sequencing data have been deposited in the NCBI Sequence Read Archive (SRA) under BioProject accession numbers PRJNA1440032 and PRJNA1440259.
