# Supplementary material for: Vitamin D receptor isoform VDRA suppresses hepatocellular tumorigenesis by restricting YAP nuclear localization
Source: Cell Death Dis. 2026 Apr 29;17(1):573. doi: 10.1038/s41419-026-08792-0 (PMC13269896; doi:10.1038/s41419-026-08792-0)
Supplement: Supplementary file 1 — Supplementary data [file 41419_2026_8792_MOESM1_ESM.docx]

**Supplementary Figures**

**Fig. S1 Expression of VDR in hepatocellular carcinoma and its impact on survival.** **(A)** The level of VDR within normal liver and hepatocellular carcinoma tissues; **(B)** Expression of VDR in tissues of different stages of hepatocellular carcinoma**; (C-D)** Disease free survival or overall survival analysis of VDR in hepatocellular carcinoma.


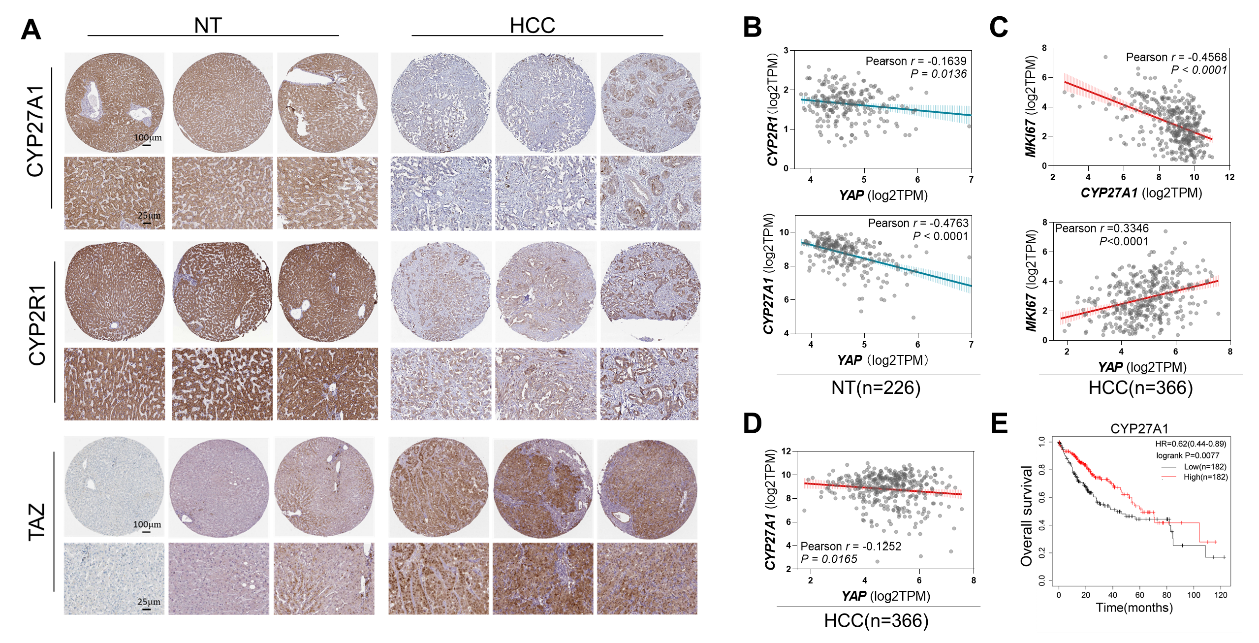


**Fig. S2 Negative correlation between 1,25(OH)_2_D_3_-actived proteins and YAP expression in human normal liver and HCC tissues.** (**A)** Immunohistochemical results of 1,25(OH)_2_D_3_-actived proteins (CYP27A1 and CYP2R1) and YAP in tissues from 3 normal livers and 3 HCC samples. (**B)** Correlation analysis of 1,25(OH)_2_D_3_-actived proteins with YAP expression in 226 healthy human liver samples, data form CTEx database. (**C)** Correlation analysis of CYP27A1 with YAP expression in 366 HCC samples (TCGA). (**D)** Correlation analysis of CYP27A1 and YAP with Ki67 expression in 366 HCC samples (TCGA). (**E)** Overall survival analysis of CYP27A1 in hepatocellular carcinoma (TCGA). Pearson correlation was used for correlation analysis, and *p*<0.05 was considered statistically significant.


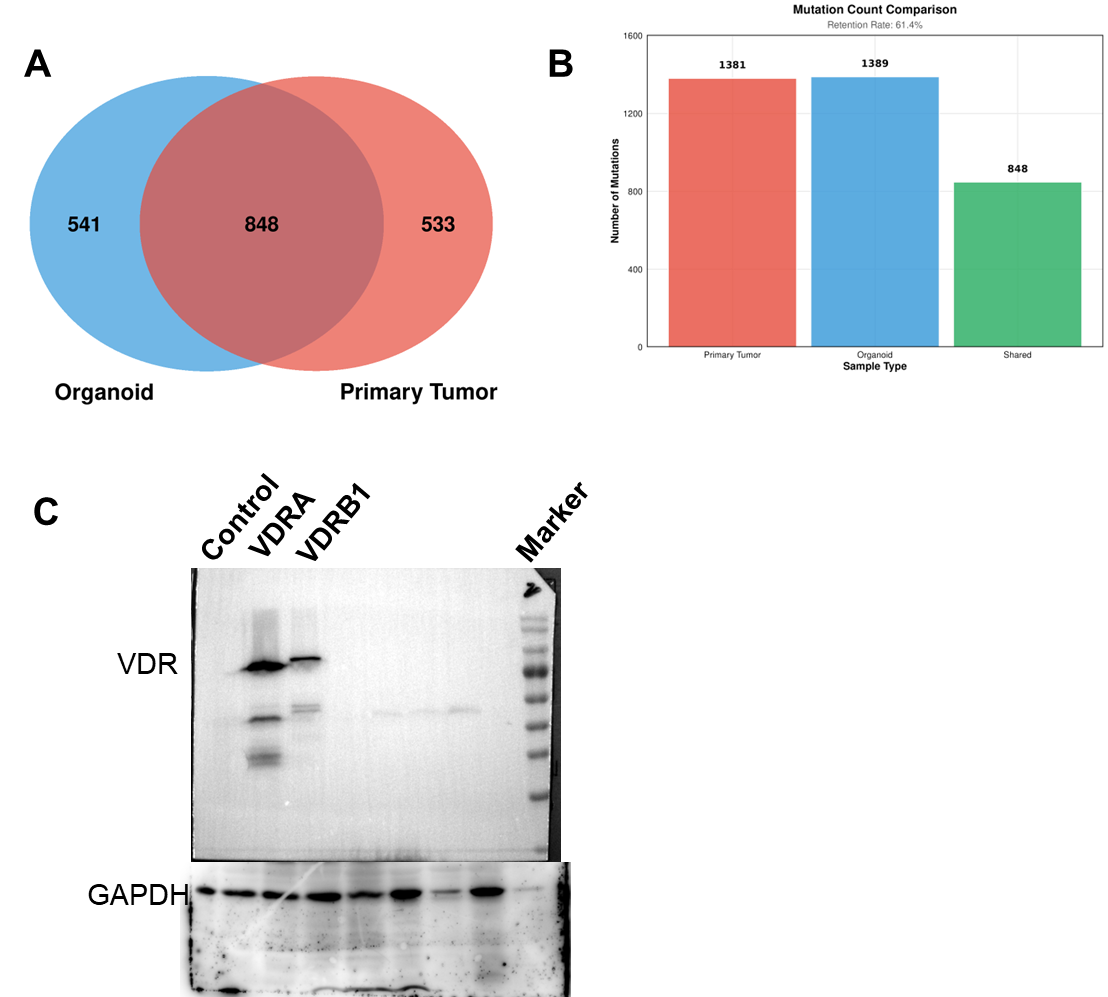


**Fig. S3 WES analysis of the somatic mutations identified in primary tumors and the matched organoids.** (**A**) Venn diagram showing the overlap of expressed genes between patient-derived organoids and matched primary tumors; (**B)** Bar plot comparing the retention rates of molecular features between organoids and primary tumors. The average retention rate was 81.4%, indicating a high degree of similarity; **(C)** Western blot analysis confirmed the overexpression of VDRA and VDRB1 in organoids.

**Supplementary Tables**

**Table S1 Immunohistochemical array human sample information**

|  | **ID** | **Sex** | **Age** | **Tissue** | **Disease** |
| --- | --- | --- | --- | --- | --- |
| **Sample#1** | #3402 | Female | 54 | Liver (T-56000) | Normal tissue, NOS (M-00100) |
| **Sample#2** | #3222 | Female | 63 | Liver (T-56000) | Normal tissue, NOS (M-00100) |
| **Sample#3** | #1720 | Male | 67 | Liver (T-56000) | Normal tissue, NOS (M-00100) |
| **Sample#4** | #3344 | Male | 67 | Liver (T-56000) | Cholangiocarcinoma (M-81603) |
| **Sample#5** | #3625 | Male | 59 | Liver (T-56000) | Cholangiocarcinoma (M-81603) |
| **Sample#6** | #4836 | Female | 64 | Liver (T-56000) | Cholangiocarcinoma (M-81603) |

**Table S2 Immunohistochemical quantification of CYP27A1**

| **Ki 67** | **Sample #1** | **Sample #2** | **Sample #3** | **Sample #4** | **Sample #5** | **Sample #6** |
| --- | --- | --- | --- | --- | --- | --- |
| **Cell types** | Hepatocytes | Hepatocytes | Hepatocytes | Hepatocytes | Hepatocytes | Hepatocytes |
| **Staining** | Not detected | Not detected | Not detected | Medium | Medium | Medium |
| **Intensity** | Negative | Negative | Negative | Moderate | Moderate | Moderate |
| **Quantity** | None | None | None | 75%-25% | >75% | 75%-25% |
| **Location** | None | None | None | Cytoplasmic/membranous | Cytoplasmic/membranous | Cytoplasmic/membranous |

**Table S3 Immunohistochemical quantification of YAP**

| **YAP** | **Sample #1** | **Sample #2** | **Sample #3** | **Sample #4** | **Sample #5** | **Sample #6** |
| --- | --- | --- | --- | --- | --- | --- |
| **Cell types** | Hepatocytes | Hepatocytes | Hepatocytes | Hepatocytes | Hepatocytes | Hepatocytes |
| **Staining** | Not detected | Not detected | Not detected | Not detected | Low | Low |
| **Intensity** | Negative | Negative | Negative | Negative | Weak | Weak |
| **Quantity** | None | None | None | None | >75% | >75% |
| **Location** | None | None | None | None | Cytoplasmic/membranous | Cytoplasmic/membranous/nuclear |

**Table S4 Immunohistochemical quantification of CYP27A1**

| **CYP27A1** | **Sample #1** | **Sample #2** | **Sample #3** | **Sample #4** | **Sample #5** | **Sample #6** |
| --- | --- | --- | --- | --- | --- | --- |
| **Cell types** | Hepatocytes | Hepatocytes | Hepatocytes | Hepatocytes | Hepatocytes | Hepatocytes |
| **Staining** | Medium | Medium | Medium | Medium | Not detected | Not detected |
| **Intensity** | Moderate | Moderate | Moderate | Moderate | Negative | Negative |
| **Quantity** | >75% | >75% | >75% | 75%-25% | None | None |
| **Location** | Cytoplasmic/membranous | Cytoplasmic/membranous | Cytoplasmic/membranous | Cytoplasmic/membranous | None | None |

**Table S5 Immunohistochemical quantification of CYP2R1**

| **CYP2R1** | **Sample #1** | **Sample #2** | **Sample #3** | **Sample #4** | **Sample #5** | **Sample #6** |
| --- | --- | --- | --- | --- | --- | --- |
| **Cell types** | Hepatocytes | Hepatocytes | Hepatocytes | Hepatocytes | Hepatocytes | Hepatocytes |
| **Staining** | High | High | High | Medium | Medium | Medium |
| **Intensity** | Strong | Strong | Strong | Moderate | Moderate | Moderate |
| **Quantity** | >75% | >75% | >75% | >75% | >75% | >75% |
| **Location** | Cytoplasmic/membranous | Cytoplasmic/membranous | Cytoplasmic/membranous | Cytoplasmic/membranous | Cytoplasmic/membranous | Cytoplasmic/membranous |

**Table S6 Immunohistochemical quantification of TAZ**

| **TAZ** | **Sample #1** | **Sample #2** | **Sample #3** | **Sample #4** | **Sample #5** | **Sample #6** |
| --- | --- | --- | --- | --- | --- | --- |
| **Cell types** | Hepatocytes | Hepatocytes | Hepatocytes | Hepatocytes | Hepatocytes | Hepatocytes |
| **Staining** | Low | Low | Low | Medium | Medium | Medium |
| **Intensity** | Moderate | Moderate | Moderate | Moderate | Moderate | Moderate |
| **Quantity** | <25% | <25% | <25% | >75% | >75% | >75% |
| **Location** | Cytoplasmic/membranous | Cytoplasmic/membranous | Cytoplasmic/membranous | Cytoplasmic/membranous/nuclear | Cytoplasmic/membranous/nuclear | Cytoplasmic/membranous/nuclear |
